# Telephone-delivered behavioral intervention among blacks with sleep apnea and metabolic syndrome: study protocol for a randomized controlled trial

**DOI:** 10.1186/1745-6215-15-225

**Published:** 2014-06-12

**Authors:** Natasha J Williams, Girardin Jean-Louis, Clinton D Brown, Samy I McFarlane, Carla Boutin-Foster, Gbenga Ogedegbe

**Affiliations:** 1 Center for Healthful Behavior Change, Division of Health and Behavior, Department of Population Health, New York University Langone Medical Center, New York University School of Medicine, 227 East 30th St, 10016 New York, NY, USA; 2 Brooklyn Health Disparities Center, Division of Cardiovascular Medicine, SUNY Downstate Medical Center, 11203 Brooklyn, NY, USA; 3 Division of Endocrinology, Department of Medicine, SUNY Downstate Medical Center, 11203 Brooklyn, NY, USA; 4 Center of Excellence in Disparities Research, Weill Cornell Medical College, 10065 New York, NY, USA

**Keywords:** Obstructive sleep apnea (OSA), Continuous positive airway pressure (CPAP), Adherence, blacks, Metabolic syndrome, Telephone-delivered, Health education, Motivational interviewing

## Abstract

**Background:**

Lack of adherence to recommended treatment for obstructive sleep apnea remains an ongoing public health challenge. Despite evidence that continuous positive airway pressure (CPAP) is effective and improves overall quality of life, adherence with the use of CPAP in certain racial/ethnic groups, especially blacks, is suboptimal. Evidence indicates that the incidence and prevalence of obstructive sleep apnea are higher among blacks, relative to whites, and blacks are less likely to adhere to recommended treatment compared with other racial/ethnic groups.

**Methods:**

Using a two-arm randomized controlled design, this study will evaluate the effectiveness of a culturally and linguistically tailored telephone-delivered intervention to promote adherence to physician-recommended sleep apnea assessment and treatment among blacks with metabolic syndrome, versus an attention-control arm. The intervention is designed to foster adherence to recommended sleep apnea care using the stages-of-change model. The intervention will be delivered entirely over the telephone. Participants in the intervention arm will receive 10 phone calls to address challenges and barriers to recommended care. Outcomes will be assessed at baseline, and at 6- and 12-months post-randomization.

**Discussion:**

This tailored behavioral intervention will improve adherence to sleep apnea assessment and treatment among blacks with metabolic syndrome. We expect to demonstrate that this intervention modality is feasible in terms of time and cost and can be replicated in populations with similar racial/ethnic backgrounds.

**Trial registration:**

The study is registered at clinicaltrials.gov NCT01946659 (February 2013)

## Background

Obstructive sleep apnea is a major public health problem in the United States, affecting an estimated 18 million Americans [1-3]. It is a serious, potentially life-threatening condition characterized by repeated cessation of breathing while sleeping. According to the National Institute of Health, sleep apnea is thought to be as prevalent as adult diabetes [4]. Others view it as big a public health hazard as smoking, heart attacks and stroke [3,5]. Using a respiratory disturbance index (referred to the total number of apneas (complete cessation of breathing lasting ≥ 10 seconds)), the Wisconsin Sleep Cohort Study estimated that sleep apnea affects as much as 15% of men and 5% of women (ages 30 to 60 years) [6]. Prevalence rates are even higher in this age group when polysomnographic criteria are used, indicating that 24% of men and 9% of women may have sleep apnea [7]. Sleep apnea is associated with obesity, hypertension (HTN), diabetes (DM), dyslipidemia, cardiovascular disease (CVD), vehicular accidents, and mortality [8]. It is estimated that sleep apnea is probably responsible for 38,000 cardiovascular deaths yearly, with an associated 42 million dollars spent on related hospitalizations [9]. Despite these alarming data, 82% of men and 93% of women with sleep apnea in the United States remain undiagnosed.

In light of available evidence, sleep apnea assessment is paramount. Standard of care for sleep apnea assessment typically entails a detailed sleep history, which is performed by a sleep clinician in a structured setting-otherwise known as a sleep laboratory. Participants who are at risk, based on subjective ratings, are routinely referred for laboratory polysomnography. A nocturnal polysomnographic study incorporates assessment of sleep architecture, airflow and ventilatory effort, arterial blood saturation, electrocardiogram, body position, and periodic limb movement [10,11].

The most effective, noninvasive treatment for sleep apnea requires the use of continuous positive airway pressure (CPAP) [12]. This treatment requires participants to wear a sealed mask over the nose, or in certain cases both the nose and mouth, while sleeping. Participants receive forced room air via the mask (that has been fitted by a technician), titrating the pressure in the oropharyngeal airway, which helps to maintain airway patency. Use of CPAP as a therapeutic modality for sleep apnea is often coupled with some form of behavior modification targeting individual’s weight [13,14].

Being male, overweight, and over the age of 40 years are risk factors for sleep apnea. Recent data strongly suggest that race/ethnicity should also be considered as an important risk factor. A home sleep study performed among community-dwelling adults (aged ≥ 65 years) showed that blacks (that is, African American, African, or Caribbean American) were 2.5 times more likely to have an apnea hypopnea index of 30 or higher compared with whites [15]. Ethnic disparities in sleep apnea estimates might even be greater if one considered a respiratory disturbance index > 10. In effect, in a study comparing 225 black and 622 white volunteers, aged 2 to 86 years, 31% of blacks versus 10% of whites had sleep apnea [16]. Another worthy observation in that study is that blacks (≤ 25 years) may be at greater risk for sleep apnea than their white counterparts [17]. In our previous work (conducted by the principal investigator (PI, Dr. Jean-Louis), we demonstrated that among blacks in Brooklyn, a history of cardiovascular disease (CVD) was the most important predictor of the likelihood of expressing symptoms of sleep apnea, with a corresponding multivariate-adjusted odds ratio of 11 (95% confidence interval = 3.03 to 40.63) [18]. Despite alarming evidence that metabolic risk markers (for example, obesity, hypertension (HTN), and diabetes mellitus (DM)) for CVD are more prevalent among blacks [19], the vast majority of suspected cases in minority populations remain undiagnosed. Under-diagnosis among blacks is of great concern since the burden of sleep apnea is worse in that population [16]. Furthermore, while adherence to CPAP therapy remains poor for the general population, with only half of participants using CPAP for more than 4 hours per night, adherence to CPAP treatment among blacks is abysmally worse. One study conducted among non-Hispanic Caucasians and non-Hispanic blacks found that CPAP use among blacks was 2.07 hours less than among whites [20]. Therefore, the development of innovative interventions for vulnerable populations, particularly for blacks, is warranted. Such interventions may have significant clinical, social and public health implications.

The primary aim of this on-going randomized trial is to assess the efficacy of a culturally and linguistically tailored telephone-delivered intervention to increase adherence to physician-recommended assessment and treatment of sleep apnea. The secondary aims are to: 1) evaluate the maintenance of intervention effects on adherence at 6 months post-randomization; and 2) assess treatment effects on indices of the metabolic syndrome (waist circumference, blood pressure (BP), lipid levels and fasting plasma glucose (FPG) or hemoglobin (HbA1c) for those who have a DM diagnosis). There are four main hypotheses for this study: 1) participants will show greater adherence to physician-recommended polysomnographic sleep apnea evaluation during follow-up assessment; 2) participants will show greater adherence to CPAP treatment during follow-up assessment; 3) effects of the intervention on adherence rates will be maintained 6 months after discontinuing active interventions, and treatments will improve indexes of the metabolic syndrome; and 4) participants’ knowledge about sleep, self-efficacy, readiness, and trust/rapport with the health educator (HE) will mediate intervention effects on study outcomes. The study is registered in clinicaltrials.gov.

## Design and methods

This is a randomized controlled trial to examine the efficacy of a culturally and linguistically tailored behavioral telephone-delivered intervention to increase adherence to physician-recommended assessment and treatment of sleep apnea. Participants will be randomized either to a tailored-telephone intervention (TTI) arm or an attention-control (AC) arm. Approximately 340 black participants at risk for sleep apnea will be randomly assigned to one of the two treatment conditions (170 in each arm). Intervention content for the TTI will be delivered by a trained HE who has experience working with minority populations. The HE has a bachelor’s degree and will receive significant training in sleep medicine, including both basic theories and laboratory-based recording and treatment procedures. She will be supervised throughout the study period by the lead investigator. Assessments will be conducted at baseline, and at 6- and 12-months post-randomization. Participants will receive $10 at baseline and $100 at the 6-month follow-up.

The institutional review boards (IRBs) at SUNY Downstate Medical Center (protocol #09-193) and NYU Langone Medical Center (protocol #13-00182) approved this study prior to implementation.

### Theoretical framework

**Figure 1:**
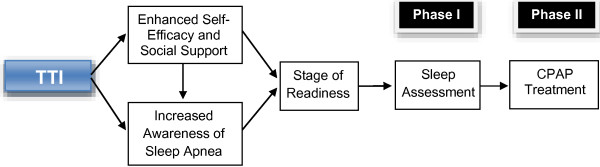
Theoretical framework for the impact of telephone interventions on adherence to recommended sleep apnea assessment and treatment.

The intervention is guided by the transtheoretical model developed by Prochaska et al. (Figure 1) [21]. This behavior change model consists of five stages that the individual moves through in the process of modifying a problem behavior. The first stage begins with pre-contemplation, in which a person is not considering change and is often unaware that they have a problem to be modified; it continues to the maintenance stage, where the individual has successfully altered the behavior (for 6 months or more) and concentrates on maintaining this new way of life. Between these two stages, the person may contemplate change, prepare to change, and begin to act to initiate behavior change. This model has been applied to a variety of health behaviors, such as alcohol and substance use, smoking cessation, condom use, and weight loss [21]. Our study will examine the relationship between participants’ readiness to use CPAP at the beginning of the intervention with the success they are likely to achieve in increasing adherence to physician-recommended sleep apnea assessment and CPAP treatment.

### Study sites/population

**Figure 2:**
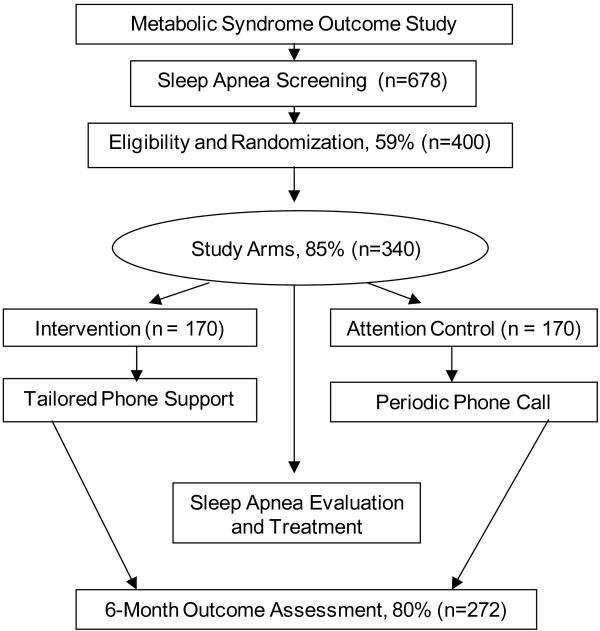
Depiction of flow of participants in the trial.

Figure 2 shows a flow chart of participant enrollment. The study sample will be drawn from a well-characterized registry of black participants of the Metabolic Syndrome Outcome Cohort Study (MetSO). A complete description of the MetSO project has been reported elsewhere [22]. Briefly, MetSO was conducted to increase access to at-risk minority participants into clinical research and intervention programs. To date, over 1,200 black participants-60% being of Caribbean origin - with cardio-metabolic diseases have been enrolled. Table 1 provides baseline characteristics of the MetSO study population. A subset of 70 participants from the registry was randomly selected to estimate risk of sleep apnea using the Apnea Risk Evaluation System (ARES™) questionnaire, which includes questions on sleep patterns, daytime functioning, and knowledge of sleep apnea [23]. Overall, the rate of sleep apnea symptoms was: snoring (72%), excessive daytime sleepiness (65%), and difficulty maintaining sleep (75%). Of the subsample, 79% reported a history of hypertension, 72% a history of heart problems, and 55% indicated having a diagnosis of diabetes. Preliminary analysis suggested a significant number of participants (59%) were at high risk for sleep apnea. Our pilot work established the infrastructure and provided estimated sample size to conduct this randomized controlled trial.

**Table 1:** Baseline characteristics of MetSO study participants

**Characteristics**	**All blacks**	**US blacks**	**Caribbean blacks**
**Age**	52.3 ± 12.3	51.17 ± 15.8	53.46 ± 11.6
**Female**	56.0%	60.8%	52.0%
**Male**	44.0%	39.2%	48.0%
**< High school**^**a**^	43.3%	31.2%	53.3%
**Hypertension**	73.1%	71.6%	76.0%
**Diabetes**	50.2%	45.6%	54.0%
**Dyslipidemia**	48.4%	42.4%	53.3%
**Obesity**	31.6%	34.9%	28.8%

^a^< High school, education up to less than high school level.

### Eligibility criteria

Eligible participants must: 1) self-report their race/ethnicity as black/African American; 2) be ≥ 18 years old; 3) diagnosis of metabolic syndrome; and 4) have a positive screening for sleep apnea, as measured by the Apnea Risk Evaluation System (ARES™) questionnaire [23]. Exclusion criteria include: 1) documented co-existing sleep problems; 2) documented illness or disability in which death is expected within two years of enrollment; 3) intent to relocate from the New York City area within one year following enrollment; 4) non-English speaking; 5) not accessible via telephone; 6) impaired cognitive or functional ability; and 7) another family member participating in the study. All participants will be asked to provide written informed consent before enrollment.

### Recruitment and screening

We aim to recruit a total of 340 black participants at risk for sleep apnea. Trained research assistants (RAs) will contact eligible participants using the MetSO registry, which includes detailed contact information. If a valid phone number was not provided, then an invitation will be mailed to the address on file. The invitation will provide instructions for contacting the project staff to receive more information about the study. Participants will be considered ineligible if the invitation is mailed to a wrong address. During the initial phone call, the RAs will provide a detailed description of the study. Participants will be contacted during the day as well as during the evening hours. Participants who express interest will be screened for eligibility. The RAs will also collect basic demographic information that will be used to assess characteristics of participants who agree to be enrolled versus those who decline solicitation to enroll in the study protocol. Eligible participants will receive a packet that includes a brief questionnaire on sleep problems, medical history, and use of medications. The packet will also include two educational brochures about sleep apnea: 'Sleep Apnea: Is Your Patient at Risk?', produced by the National Heart Lung and Blood Institute and 'Sleep Apnea', produced by the American Academy of Sleep Medicine (AASM).

### Randomization

Upon completion of the baseline interview, consenting participants will be randomly assigned to either the TTI or the AC arms. Randomization will be conducted by the statistician and will be carried out in pairs using a table of permutations [24]. This strategy will ensure balance between the two study arms regarding factors including participant characteristics, lifestyle behavior, and medical comorbidities. Due to the nature of the study, the HE delivering the intervention will not be blind to study arms. After completion of the 12-month assessment, all AC participants will be invited to participate in the TTI. Based on our experience working with this community, we believe that failure to offer the TTI could severely jeopardize community relations.

### Description of the tailored telephone intervention

The intervention is culturally and linguistically tailored to address the needs of black participants. We believe that interventions should take into consideration the unique cultural and ethnic norms and values of the target population in order to have a greater likelihood of success. Before implementation, we conducted formative research, including focus groups with the target population during which the TTI materials were pilot tested. Study investigators also relied on a community advisory board to provide input on the intervention design, including program materials for specific content areas, such as appropriate messaging and educational levels.

The intervention will be delivered entirely over the telephone by a trained HE. It is based on evidence that telephone-delivered interventions are effective for increasing rates of screening and treatment for various health problems, including colorectal cancer [25-27] and depression [28], appointment keeping [29], and adherence to treatment [30,31]. In addition, it is advantageous because it is cost-effective and participants do not need to leave their home. Using the transtheoretical model, each phone contact will be individually tailored to assess stage of readiness as well as the participant’s level of motivation. In pre-contemplation, communications will attempt to increase motivation to change. Further along the continuum, communications will facilitate acting on motivation and maintaining a behavior. For participants with low levels of motivation, the goal will be to increase motivation and commitment; while for those who already appear motivated, motivation will be enhanced by increasing knowledge, modifying beliefs, and increasing the value placed on sleep assessment.

### Phase I - sleep apnea assessment

Within 2 weeks following randomization, the HE will contact participants assigned to the TTI arm by telephone using a detailed script to guide each phone contact. The goals of the first telephone contact will be to obtain a detailed assessment of the participant’s knowledge, attitudes, and behaviors related to sleep and to improve understanding of sleep apnea. The HE will emphasize the need for participants to undergo a sleep apnea assessment at a local sleep center. The HE will also provide participant navigation and identify logistical barriers to receiving the sleep assessment, such as transportation, and intervene as necessary. During this phone contact the HE will assess participants’ stage of readiness to change and will counsel the participant accordingly. Once a verbal commitment to schedule a sleep assessment is established, the HE will record the date and time of the appointment and will follow up with participants the day after to determine if the appointment was kept. If participants do not keep the appointment the HE will identify obstacles and will work with participants to achieve the intended goal. In cases where participants keep the appointment, the HE will inquire about the outcome of the assessment, including whether or not there was a diagnosis of sleep apnea. For participants with a confirmed diagnosis of sleep apnea, the emphasis will be on treatment for sleep apnea (see 'Phase II - adherence to CPAP treatment and maintenance' below). The HE will be instructed to make up to 10 phone calls, each lasting up to 25 minutes with an average of 10 minutes per call. The HE will keep implementation logs. These will contain date and time of attempted and completed calls, length of call, participant’s level of commitment to receive an assessment and follow-up care, participant’s self-report of obtaining the assessment and follow-up, type of barriers encountered and ways to overcome them, and time spent discussing various topics (for example, sleep apnea, other health-related topics, personal issues).

### Phase II - adherence to CPAP treatment and maintenance

During phase II, emphasis will be on CPAP adherence where the HE will work with participants to facilitate acceptance and adherence to prescribed CPAP treatment. Similar to phase I, a standardized intervention script will be used. The HE will work with the participants to identify barriers and focus on strategies for maintaining treatment and behavior change. In addition to receiving continued support and education, participants will be instructed about the importance of receiving the appropriate follow-up care at the sleep center. During these phone contacts the HE will emphasize that sleep apnea is a chronic condition that requires ongoing self-management.

### Follow-up procedures

Participants will be instructed that appropriate management of their condition may require attending approximately three clinic visits at the sleep center; the number of visits will vary based on the participants’ diagnosis. During the first visit to the sleep center, participants will have a consultation with the sleep specialist, and complete questionnaires. The sleep specialist will conduct an overview of the participant’s sleep history, and medical history including BP, blood glucose and other health behaviors (for example, regularity of physical examinations, cholesterol screening, smoking and drinking habits, and exercise habits) will be assessed. This initial consultation will also provide participants with another opportunity to ask any questions pertaining to the study procedures and to help participants feel comfortable with the testing environment. Upon diagnosis of sleep apnea, participants will complete a follow-up visit that will include an overnight CPAP titration study. Participants will receive additional information on how to use the device and participants will be reminded that the HE will be available to answer any further questions.

### Description of the attention-control arm

The standard of care for adults with sleep apnea includes a referral for a comprehensive assessment of sleep apnea and provision of standard print communications about sleep apnea produced by the National Institutes of Health and the AASM. Participants in the control arm will receive up to 10 phone calls from a RA to provide information about sleep apnea and information about available resources. During these phone calls, no tailored behavioral change strategies will be used. The periodic phone calls are consistent with current practice of many medical facilities that have adopted the policy of making multiple phone calls to remind participants of scheduled doctor’s appointments. In addition, instituting this procedure ensures that differences in adherence rates are attributed solely to the delivery of the intervention.

### Exit interviews

Finally, all participants will be debriefed. Participants will complete the same baseline questionnaires and additional questionnaires designed to understand why participants adhered to the intervention and how the intervention might be refined to be more effective.

### Data safety and monitoring

In compliance with the National Institutes of Health regulations, an internal data and safety monitoring committee has been developed. The purpose of the committee is to ensure the safety of participants and the validity and integrity of the data. The committee will meet once per year to monitor the overall conduct of the study. In addition, all participants will be subject to a chart audit and participants will undergo physical exams before and after the intervention. During both visits, participants will be asked to bring bottles of any medications they are currently taking, including statins, antihypertensive, antihyperglycemic, and sleep medications (prescribed and/or over the counter). Trained RAs will note each medication, its name, drug class, and the doses at baseline. During follow-up visits, the RAs will note if there are any changes to any of the medications noted at the previous visit and if a new medication has been added. Participants will be monitored during the course of data acquisition and any health concerns that may arise will be addressed, particularly among participants with no identifiable primary-care physicians, in which referrals will be made to primary-care clinics.

### Treatment fidelity

A comprehensive plan for training the HE was essential to the development of the intervention. The training curriculum includes an overview of sleep medicine, the recommended AASM guidelines, stages of readiness, and cultural sensitivity. Prior to recruitment, the HE received 30 hours of training in sleep medicine. The HE will be continuously monitored throughout the study period to ensure adherence to study protocol and ascertain knowledge and application of the AASM obstructive sleep apnea assessment and treatment guidelines. At the beginning of the study, the HE was required to attend workshops on cultural sensitivity. Moreover, the HE was selected based on her experience working with minority populations. The HE received additional training in individual tailoring of intervention approaches based on participants’ stage of readiness. During the training, the HE was observed implementing the intervention protocol and sample activities. This allowed the HEs to receive feedback from the PI and team members who suggested ways to overcome challenges and barriers. Finally, the HE will meet with the study PI on a bi-monthly basis for a progress report, discussing challenges encountered during the TTI delivery.

### Qualitative evaluation

Another unique component of this study is that we integrated qualitative research with the randomized controlled trial design. At the completion of the trial we will conduct focus groups to assess participants’ overall impressions of the intervention. More specifically, we will conduct four focus groups (two with each arm) with a sample of eight to ten participants in each group 6 months after completion of the study to ascertain the following: what did participants like and not like about the intervention? How could it have been done better? Which components were most responsible for promoting changes? Why did some seek an assessment of sleep apnea (and used CPAP) while others did not? Which intervention components were most and least acceptable? Were there any critical incidents that prompted respondents to get a sleep apnea assessment? How could the intervention be changed to make it more effective? A semi-structured interview guide will be developed based on our formative research conducted prior to the start of the intervention.

### Measures

#### Sociodemographic variables

Sociodemographic variables will include age, type of health insurance, marital status, education, place of birth (for example, US versus Caribbean), years in current residence and locality, number of children living in household, number of adults living in household, self-described ethnicity, and annual household income.

#### Apnea-risk evaluation system questionnaire

This tool includes questions on sleep patterns, daytime functioning, knowledge of sleep apnea, and diseases associated with sleep apnea (that is, HTN, DM, heart disease) [23].

#### Epworth sleepiness scale

This questionnaire uses a Likert-type scale in which the respondent indicates the most appropriate number ranging from 0 = would never doze or sleep to 3 = high chance of dozing or sleeping based on a given situation. A score of 10 or more is considered excessive sleepiness [32].

#### Apnea knowledge test and apnea beliefs scale

These two questionnaires assess participants’ understanding and beliefs of obstructive sleep apnea and CPAP treatment. The AKT includes 13 questions with a yes/no response choice and 2 open-ended questions; the ABS includes 24 items on a five-point Likert scale ranging from 'strongly agree' to 'strongly disagree'. A higher score on the AKT indicates better knowledge and understanding of the illness and treatment and a higher score on the ABS indicates greater likelihood of adherence to treatment [33].

#### Change assessment scale

This is a 32-item instrument that assesses a person’s readiness to change. Participants evaluate the extent to which they strongly agree or strongly disagree with various statements such as, 'I’ve been thinking that I might want to change something about myself' and 'I have problems and I really think I should work on them' [34].

#### Medical history

Medical history includes family history of sleep disorders, obesity, DM, HTN, dyslipidemia, and CVD; history of other preventive behaviors (for example, prostate exams), and other health behaviors (for example, regularity of physical examinations, cholesterol screening, smoking and drinking habits, and exercise habits).

#### Adherence to CPAP

We will assess CPAP adherence using subjective and objective measures. A self-report scale will be used to track CPAP adherence at the end of the 6-month period. CPAP adherence is defined as the mean number of hours per day and days per week participants report using CPAP [35,36] (adherence: > 4 hours a night for 70% of the nights or no report of symptomatic complaints). Throughout the study, CPAP adherence data will be electronically monitored on a daily basis. Data will be downloaded directly from CPAP devices [37].

A complete list of measures and the data collection schedule is provided in Table 2.

**Table 2:** Schedule to acquire study data

**Outcome measures**	**Baseline**	**First follow-up**^**a**^	**Second follow-up**
Sociodemographic and clinical variables	X		
Apnea-risk evaluation system questionnaire	X		
Epworth sleepiness scale		X	X
CES-D	X	X	
Apnea knowledge test	X	X	
Apnea beliefs scale	X	X	
Medical outcomes study short form 36	X		
Self-efficacy scale	X	X	
Change assessment scale	X		
Functional outcomes in sleep questionnaire	X	X	
Process variables	X	X	
*Post hoc* evaluation variables			X
Sleep variables (chart audit)		X	

^a^Follow-up outcome ascertainment is performed by the Data Manager at 6 months post-enrollment and at 6 months post-telephone intervention (TTI).

Cardio-metabolic measures are as follows. (1) Weight will be measured without shoes and using a validated digital scale. (2) Waist circumference will be measured by 1) locating the upper hip bone and the top of the right iliac crest, 2) placing the measuring tape horizontally around the abdomen at the level of the iliac crest (tape is snug without causing compression), and 3) measuring at the end of a normal expiration. (3) BP measurements will be taken following American Heart Association guidelines [38]. Using a BPTru automated BP monitor, five readings will be obtained while participants are seated comfortably for five minutes prior to measurement [39]; the average BP will be used as the BP index for each visit. (4) Following the National Cholesterol Education Program standard guidelines for total cholesterol measurement, we will use the Cholestech LDX Analyzer; whole blood from fingersticks will be used for the quantitative determination of HDL-C and FPG utilizing established formulation [40]. All measurements will be collected by trained clinical research staff.

### Outcomes

The primary outcome will be adherence to physician-recommended assessment and treatment for sleep apnea. The secondary outcomes will include 1) maintenance of recommended treatment at 6 months and at 12 months; 2) waist circumference; 3) lipid levels; 4) BP; and 5) FPG or HbA1c for those who have a DM diagnosis. Outcomes assessment will be conducted at baseline, and at 6- and 12-months post-randomization.

### Chart audit

Upon completion of a sleep assessment, a participant’s chart (intervention or attention control) will be subjected to a random audit. Audits for confirming self-report will be conducted by one of the study investigators, who will be blinded to randomization status. Verification of a sleep assessment entails documentation of an appropriate sleep history and a polysomnographic study.

### Statistical analysis

To describe the characteristics of the study population, we will use measures of central tendencies and dispersion for continuous data; frequency distributions will be used for categorical data. Chi-square tests will be used to compare categorical data, and ANOVA will be used for continuous data. Relationships between mediators of adherence to recommended sleep apnea assessment and CPAP treatment will be examined using binary multivariate logistic regression analysis. The two main dependent measures will be: adherence to physician-recommended sleep assessment (yes versus no) and adherence to CPAP treatment (yes versus no). Candidate mediators will include sleep apnea knowledge, self-efficacy, readiness, and trust/rapport with the HE. Effects of sociodemographic and clinical factors will be adjusted in those analyses. Repeated-measures MANCOVA will be used to examine differences between baseline and follow-up measures (for example, waist circumference (WC), BP, HDL-C, FBG/HbA1c); age, sex, and sleepiness will be entered as covariates. MANCOVA is a robust technique that incorporates routines to account for heterogeneous variances, serial correlations, and variance inflation [41].

### Hypothesis 1

TTI will lead to higher rates of sleep apnea assessment at 6 months. A comprehensive assessment of sleep apnea will be defined by documentation of an appropriate medical and sleep history by a sleep clinician and a nocturnal polysomnographic study.

#### Analysis for hypothesis 1

Analysis will be performed on an intention-to-treat basis. All participants in the treatment arm will be assumed to have received the full dose of the telephone intervention and all participants in the attention-control arm will be assumed to have received no tailored intervention. Prior to testing for intervention effects, we will verify the comparability of the treatment and control arms with respect to all available demographic and personal data. T-tests and chi-square tests will be used for continuous and categorical variables, respectively. Thus, receipt of sleep apnea assessments within the 6-month period will be a binary outcome, yes or no. We will use logistic regression to estimate the odds associated with receipt of sleep apnea assessment contrasting the two study arms.

### Hypothesis 2

TTI will lead to higher adherence rates to recommended follow-up care (that is, CPAP use (> 4 hours a night for 70% of the nights)) post-diagnosis of sleep apnea.

#### Analysis for hypothesis 2

The analysis plan is similar to that for hypothesis 1. The focus will be on adherence to recommended CPAP treatment. We will use logistic regression to estimate the likelihood of adhering to prescribed CPAP treatment after a diagnosis of sleep apnea was made.

### Hypothesis 3

Effects of the intervention in maintaining adherence will be sustained 6 months after active interventions and interventions will improve clinical outcome measures (WC, BP, HDL-C, and FPG/HbA1c).

#### Analysis for hypothesis 3

We will test the hypothesis that participants in the intervention arm will sustain gains from the initial interactions with the HE, leading to long-term adherence to recommended treatment and follow-up care. It is routinely recommended that participants with sleep apnea return for a follow-up visit after initiating treatments, during which adherence to treatment plan and improvement in sleep parameters is assessed. In our protocol, clinical outcomes (WC, BP, HDL-C, and FPG/HbA1c) will be ascertained post treatment. The analysis plan to test adherence at 6 months post-randomization is similar as for hypotheses 1 and 2; participants returning for a follow-up visit will be grouped as long-term adherents versus non-adherents. Chi-square tests and logistic regression analyses will be used. Repeated-measures MANCOVA will be employed to examine differences between baseline and post-intervention measures (WC, BP, HDL-C, and FPB/HbA1c). *Post hoc* comparisons between groups will be performed with Bonferroni corrections, where univariate results are significant. As required by assumptions governing the use of ANOVA, and since we expect the dependent variables to be modestly correlated, we will first examine their collinearity. Next, normality of the distribution of each measure will be examined and transformations will be performed for non-gaussian distributions. In exploratory analyses, we will determine whether mixed effect modeling would support ANOVA results.

### Power and sample size calculations

Data obtained from a random sample of black participants recruited from the MetSO Cohort Study has provided a current estimate of the rate of comprehensive assessment of sleep apnea to be expected in the proposed study. Analysis of the data suggested that 47% of participants referred for sleep assessment would adhere to physician recommendations after exposure to TTI, whereas 35% of those in a control group agreed to undergo a sleep assessment. Thus, this represents the likely rate of adherence expected in our AC group. We propose to achieve an adherence rate of 47% among participants exposed to the TTI.

With an expected 12% increase in the adherence rate associated with the TTI, a standard error of 0.10%, and a sample size of 340, the study will be adequately powered (90%) to detect significant increase in the outcome measures using chi-square tests. The primary study outcome in this project is documented receipt of a sleep apnea assessment. A two-tailed test, with alpha = 0.05, comparing adherence rates of 35% with 47% requires 170 participants per group [42]. The precision estimated in that analysis is the approximate expected precision of study data. Specifically, a difference of 12% would be observed at chi-square = 21.03 with a 95.0% confidence interval of 3% to 20%. With the proposed sample size, the study will have 85% power to achieve a one-standard deviation increase in the odds ratio of adherence attributable to proposed mediators: sleep apnea knowledge, self-efficacy, readiness, and trust/rapport with the HE (expressed as continuous variables).

### Qualitative analysis

For the qualitative analysis, focus groups will be recorded and transcribed verbatim. Field notes from the HE will also be collected and reviewed. Transcripts will be reviewed line-by-line and assigned codes or labels. Similar codes will be grouped into higher order concepts and then categories. Similar categories will be clustered to form overarching themes. These themes will be the major headings describing barriers to adherence with appointment keeping and using CPAP. A detailed codebook of the analytic process will be maintained. Two independently trained RAs will review the transcripts and serve as corroborators. Data analysis will be aided by (Nvivo) software [43,44].

## Discussion

The intervention is designed to be practical so that, if efficacy is demonstrated, it can be widely disseminated. The intervention relies on one-to-one personal contact, a proven method to effect behavioral change, particularly in populations with low levels of literacy and trust. We expect to demonstrate that TTI is feasible in terms of time and cost.

The significance of the intervention lies in its ability to address a public health need in a high-risk population and minimize health disparities, to evaluate an intervention that can reduce sleep apnea-related morbidity in the community, and to improve understanding about how such interventions can be effectively implemented in other settings.

## Trial status

The MetSO trial began in January 2010. To date a total of 311 participants have been randomized to receive either TTI or usual care.

## Abbreviations

AC: attention control; BP: blood pressure; CPAP: continuous positive airway pressure; CVD: cardiovascular disease; DM: diabetes mellitus; FPG: fasting plasma glucose; HE: health educator; HTN: hypertension; MetSO: Metabolic Syndrome Outcome Cohort Study; PI: principal investigator; RA: research assistant; TTI: tailored telephone intervention; WC: waist circumference.

## Competing interests

The authors declare that they have no conflict of interest to disclose.

## Authors’ contributions

GJL, CDB, SM, CBF, GO, and NJW: conception and design of manuscript. GJL CDB, SM, and GO: financial support. NJW and GJL: drafting the manuscript. CDB, SM, CBF, and GO: revising manuscript critically for important intellectual content. All authors read and approved the final manuscript.
